# Polyphenols and ω-3 PUFAs: Beneficial Outcomes to Obesity and Its Related Metabolic Diseases

**DOI:** 10.3389/fnut.2021.781622

**Published:** 2022-01-17

**Authors:** Thais Keiko Siroma, David Johane Machate, Verônica Assalin Zorgetto-Pinheiro, Priscila Silva Figueiredo, Gabriela Marcelino, Priscila Aiko Hiane, Danielle Bogo, Arnildo Pott, Elenir Rose Jardim Cury, Rita de Cássia Avellaneda Guimarães, Marcelo Luiz Brandão Vilela, Rosângela dos Santos Ferreira, Valter Aragão do Nascimento

**Affiliations:** ^1^Graduate Program in Health and Development in the Central-West Region, Federal University of Mato Grosso do Sul, Campo Grande, Brazil; ^2^Spectroscopy and Bioinformatics Applied Biodiversity and Health - GEBABS, Federal University of Mato Grosso do Sul, Campo Grande, Brazil; ^3^Graduate Program in Materials Science, Federal University of Mato Grosso do Sul, Campo Grande, Brazil; ^4^Graduate Program in Biotechnology and Biodiversity in the Central-West Region, Federal University of Mato Grosso do Sul, Campo Grande, Brazil; ^5^Medical School, Federal University of Mato Grosso do Sul, Campo Grande, Brazil; ^6^Graduate Program in Biotechnology, S-Inova Biotech, Catholic University Dom Bosco-UCDB, Campo Grande, Brazil

**Keywords:** vegetable foodstuffs, fish foodstuff, metabolic diseases, α-linolenic acid, eicosapentaenoic acid, docosahexaenoic acid

## Abstract

Obesity is associated with the leading causes of death in the worldwide. On the other hand, the intake of vegetables, fruits and fish is related to the reduction of obesity and other metabolic syndromes. This review aims to highlight the role of ingestion of polyphenols and omega-3 polyunsaturated fatty acids (ω-3 PUFAs) in reducing obesity and related metabolic diseases (RMDs). The consumption of vegetables, fish and by-products rich in polyphenols and α-linolenic acid (ALA), as well as oils rich in eicosapentaenoic acid (EPA) and docosahexaenoic acid (DHA) are associated with a decrease in obesity and its RMDs in consumers. Furthermore, we discussed the adequate amount of extracts, powder, polyphenols, ω-3 PUFAs administrated in animal models and human subjects, and the relevant outcomes obtained. Thus, we appeal to the research institutions and departments of the Ministries of Health in each country to develop a food education joint project to help schools, businesses and families with the aim of reducing obesity and other metabolic diseases.

## Introduction

Obesity is an abnormal accumulation of fat in cells that interferes with the maintenance of an individual's health. It is a chronic disease characterized by lower amounts of energy expenditure than ingestion, leading to body weight gain over time due to excessive increase in adipose tissue mass (1), triggering pro-inflammatory agents (2). Furthermore, obesity is linked with several diseases such as insulin resistance, systematic inflammation, diabetes mellitus (DM), hypertension, coronary heart diseases (CHD), adipocyte hypertrophy, non-alcoholic fatty liver disease (NAFLD), and others (3, 4). Weight can be calculated from the mathematical formula of the body mass index (BMI = mass/height × height), being considered overweight that can progress to obesity when BMI ≥ 25 and ≥ 30 kg/m^2^ (1, 2). In the adult population, the occurrence of obesity and overweight is 39 and 50%, and it is mainly explained by the easy access to high-calorie foods (fast food) and sedentary lifestyle (5).

**Figure 1:**
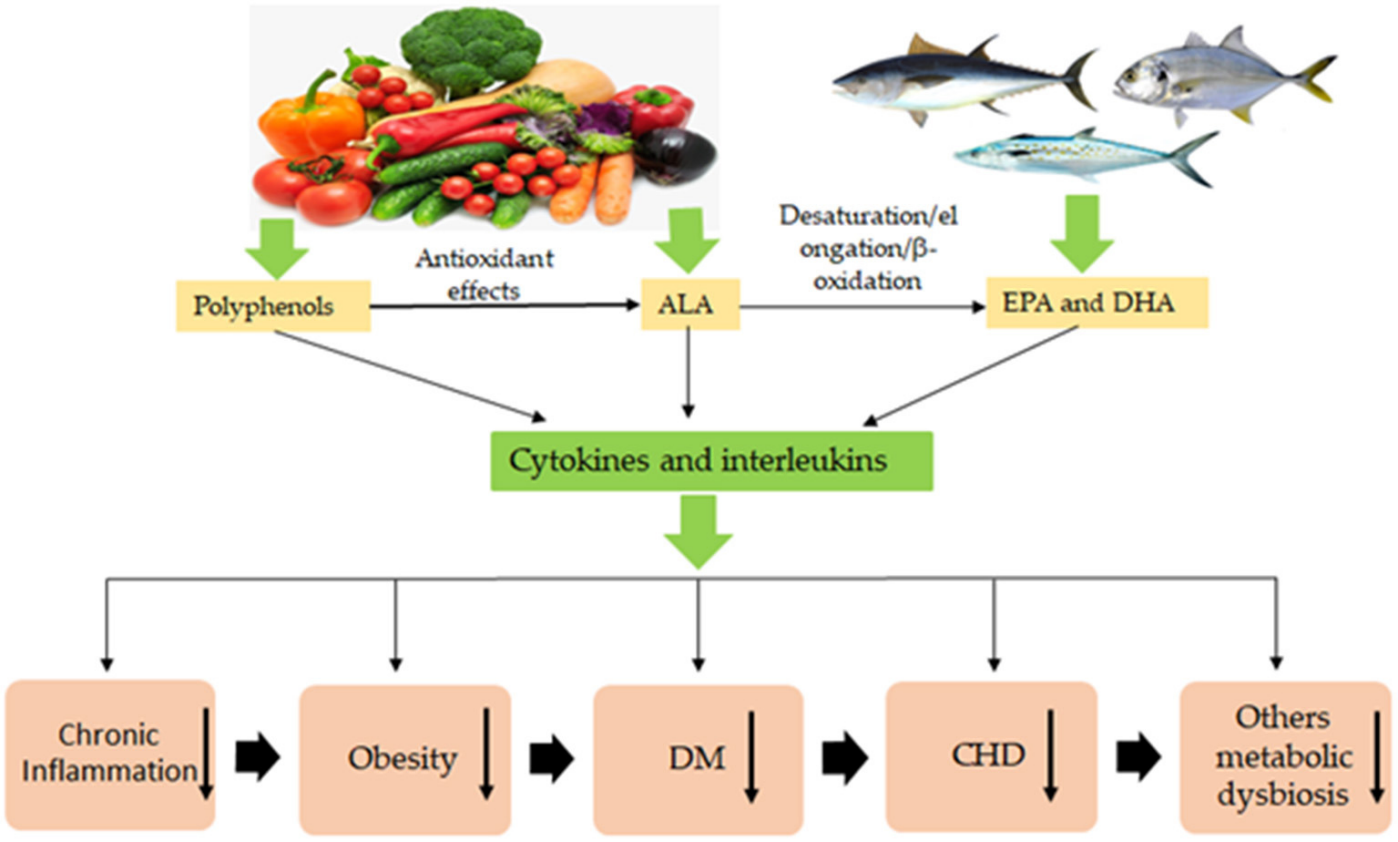
Overview of polyphenols, α-linolenic (ALA), eicosapentaenoic (EPA), and docosahexaenoic acids (DHA) natural sources. The polyphenol compounds obtained from vegetables are active natural antioxidants, which slow up or reduce the high speed of degradation of ALA, EPA, and DHA, quenching singlet oxygen and reacting or eliminating the free radicals, prolong the half-life of these acids during their storing and confection of food. The ingestion of polyphenols, ALA, EPA, and DHA in natural conditions prevent obesity and its related metabolic diseases, including these presented in the scheme. However, the benefit does not occur when polyphenols, ALA, EPA, and DHA are denaturated during the extraction process, storage, and food confection. Through the biosynthesis processes with the actions of enzymes, ALA is converted to EPA and DHA. The synergistic effects of polyphenols, EPA and DHA in the body promote health with preventing and reducing obesity and its related diseases for the consumers. ↓, significant decrease; DM, diabetes mellitus; CHD, cardiovascular heart diseases.

Systemic complications in obese patients are associated with increased abdominal fat, severe organ and tissue failure due to an increased pro-inflammatory cytokine storm, lipopolysaccharide and oxidative stress conditions (6). In addition, several studies have reported a decrease in obesity and its RMDs due to consumption of vegetables (leaves, seeds, nuts, fruits, vegetable oils, by-products) and fish (mainly marine fish, oils, by-products) rich in polyphenols and ω-3 PUFAs: ALA, EPA, and DHA (7–13). Furthermore, obesity and its RMDs lowering can be explained by consequence of synergistic actions of polyphenols and ω-3 PUFAs improving several metabolic health pathways (14, 15). Due to the synergistic actions of the polyphenols and ω-3 PUFAs, some products that are found, like fish and vegetables, and their by-products can potentially improve and control obesity and its RMDs as anti-glucose tolerance, anti-oxidative, anti-atherosclerosis, anti-inflammation, anti-weight gain, hepato-protective, vascular-protective, cardiovascular-protective, anti-hypertension, anti-diabetic effects, thus improving the human health (13, 16–21). The beneficial effects of foods that contain polyphenols, ALA, EPA and DHA in their composition are summarized in Figure 1.

However, despite the reported benefit of polyphenols and ω-3 PUFAs reported, obesity and its RMDs high incidence can be correlated with inadequate food intake (22), the lower cost of unhealthy food acquisition (23) and cultural behaviors barriers (24) allied to unfavorable educational programs impact negatively on healthy food acquisition (25).

In this review, we aimed to emphasize the benefit of polyphenols and ω-3 PUFAs regular intake and their sources and to propose joint actions allied to consumer's behavior change for reducing obesity and its RMDs (systematic inflammation, cardiovascular diseases, hypertension, diabetes mellitus, high insulin level, metabolic syndrome, and others).

## The Main Polyphenols Sources

Vegetables, fruits, seeds, almonds, and cereals are widely known in diets and supplementations for their enormous benefits on health improving, preventing, and reducing obesity and its RMDs (9, 13). Health benefits are associated with the effect of bioactive substances, mainly represented by compounds with antioxidant action that are responsible for functions such as the half-life of products and their by-products (residue products as peel, pulp and seed) (12, 16, 26–30). The main polyphenol substances occurs in leaves, flowers, roots, bulbs, and rhizomes of several wild edible plants (31, 32). In addition, polyphenol is present in fruits as apple, grapes, pear, cherries, berries, coffee, cereals and chocolate (33), citrus, mangoes, garlic, onions (34), tomatoes, potatoes, carrots, leaves (tea), and vegetables (broccoli, cabbages, pumpkin, spinach, and lettuce). In addition, these plants (35) are natural sources of anthocyanins and stilbenes (resveratrol and piceatannol) (26), catechin, quercetin, kaempferol (27), umbelliferone, epicatechin, phenolic acids (gallic, ellagic, chlorogenic, caffeic, and coumaric) (34), hydroxytyrosol, tyrosol (35), curcumin, rutin, chrysin (36), myricetin, isorhamnetin, hesperidin, narirutin, naringin, apigenin, luteolin, pelargonidin, cyanidin, delphinidin, genistein, daidzein (37), ellagitannins, and others (38). The effects of vegetables, fruits and polyphenols on obesity and its RMDs in animal models are summarized in Table 1.

**Table 1:** Effects of polyphenols intake on obesity and its related metabolic diseases outcomes in animal model.

**Vegetable/fruit**	**Host**	**Diet**	**Main outcomes**
*Camellia sinensis* (Tea)	Mice ICR (7 weeks old) male obese (39)	Six leaf drinking tea types: green, black, yellow, white, oolong and post- fermented (13–15 g/kg/day) for 9 weeks	Body weight ↓ White fat ↓ Hepatic steatosis ↓ Obesity effects ↓ Anti-inflammatory ↑ IL-6 ↓ iNOS ↓
*Vitis vinifera* (Grape)	Mice C57BL/6J (12 weeks old) obese (40)	Grape powder (23 g/kg/day) for 18 weeks	Inflammation ↓ Adipocyte tissue↓
		Grape powder extract (150 mg/kg/day) for 18 weeks	Inflammation ↓ Glucose tolerance ↓
	Wistar rats (5 weeks old) male obese (41)	Grape seed proanthocyanidin extract (25 mg/kg body weight/day) for 3 weeks	Adipocyte number ↑ Body weight ↔ Adipose tissue ↔ Adipocyte size ↓
	Wistar rats albino male diabetic (42)	Grape seed extract (50 mg/kg/day) for 3 weeks	Blood glucose ↓ Cholesterol ↓ Inflammation ↓ Hyperglycemia ↓ DM ↓
*Bactris setosa* (Tucum) and *Vitex cymosa* (Tarumã)	Mice C57BL/6J (5 weeks old) male diabetic (43)	Extract (100 mg/kg/day) for 8 weeks	Obesity ↓ Insulin resistant ↓ Hyperinsulinemia ↓
*Adansonia digitata* (Baobab)	Wistar albino rats (8 weeks old) diabetic (44)	Extract (200 and 400 mg/kg/day) for 6 weeks	HDL-c ↔ Adipose tissue↓ Diabetic ↓
*Olea europaea* (Olive)	Wistar Kyoto rats (8 weeks old) hypertensive (45)	EVOO (759 mg/kg/day) for 10 weeks	Blood pressure ↓ Cardiac hypertrophy ↓ AEF ↑ TC ↓ Pro-inflammatory ↔
	Mice C57BL/6J (5 weeks old) male diabetic (46)	EVOO (447 mg/L/day) for 24 weeks	Pro-inflammatory ↔ β-cell apoptosis ↓ β-cell number ↑ Insulin resistance ↑ Islet glucose ↑ Glucose homeostasis ↑
*Curcuma longa* (Turmeric)	Mice C57BL/6J (3–5 weeks old) male obese—diabetes (47)	Extract (0.03 mg/kg/day) for 6 weeks	Adiponectin ↑ HNF-kB ↓ Inflammation ↓ Obesity ↓
*Solanum lycopersicum* (Tomato)	Mice C57BL/6N (4 weeks old) male obese (48)	Vinegar beverage (14 mL/kg/day) for 6 weeks	Obesity ↓ Insulin resistance ↓
*Euterpe oleracea* (Açai)	Mice C57BL/6 (4 weeks old) male obese (49)	Seed extract (300 mg/kg/day) for 12 weeks	Obesity↓ Adipose tissue↓ NAFLD ↓ Cholesterol ↓
*Coffea arabica* (Coffee)	Wistar rats (8–9 week old) male obese (50)	Coffee extract (5 mg/kg/day) for 8 weeks	Obesity ↑ Cardiovascular ↓ Hepatic dysfunction ↓ Hypertension ↓
*Malus domestica* (Apple)	Wistar rats male obese (51)	Apple polyphenols (146 mg/kg) for 8 weeks	Adipose tissue ↓ Glucose tolerance ↓ Obesity ↓ Fatty acid oxidation ↑ Leptin level ↓
*Tamarindus indica* (Tamarind)	Sprague-Dawley rats (12 weeks old) male obese (52)	Tamarind fruit extract (50 mg/kg/day) for 10 weeks	Obesity ↓ Leptin ↓ Antioxidant ↑ Lipid metabolism ↔
*Brassica oleracea* var. *italica* (Broccoli)	Wistar rats (6–8 weeks old) male obese (53)	Broccoli extract (14 mg/kg/day) for 10 weeks	Body weight ↓ Adipose tissue ↓ NAFLD ↓

*↑, significant increase; ↔, unchanged; ↓, significant decrease; IL-6, interleukin- 6; iNOS, inducible nitic oxide synthase; TC, total cholesterol; DM, diabetes mellitus; HDL-c, high-density lipoprotein cholesterol; HNF-kB, Hepatic nuclear factor-kB; NAFLD, non-alcoholic fatty liver disease; AEF, aortic endothelial function; EVOO, extra virgin olive oil*.

Several studies have been reported on obesity and its RMDs lowering using different extracts from leaves, flowers, fruits, seeds, rhizome, powder, and EVOO obtained from wild and cultivated plants regularly administrated at 3 mg/kg/day to 23 g/kg/day for 3–24 weeks to animal models (Table 1). Controversially, other studies using dairy dose administrated from the green tea polyphenols (10–29 mg/kg), catechin (200 and 400 mg/kg) (54), caffeic acid, quercetin (2 or 4%) (55), and proanthocyanin grape seed extract (4 g/kg/2 weeks) (56), reported liver, kidney and gastrointestinal toxicity, which can evolve to inflammation or death, due to high reactive oxygen species and oxidative stress formation. In addition, some studies with humans administered polyphenols showed the same results that can be explained by genetic effects, ethnicity, gender, eating habits, length of time, lifestyle, and others (57). Therefore, the reported high health benefits of regular consumption of polyphenol-rich plants and vegetables are widely recommended to prevent, control and reduce obesity and RMDs in humans and animals (57). Likewise, the health benefit for humans with obesity, that administered vegetables, fruits and polyphenols for 4–12 weeks are summarized in Table 2.

**Table 2:** Effects of polyphenols from vegetables and fruits intake on obesity and its related metabolic diseases outcomes in human subjects.

**Vegetable/fruit**	**Host**	**Diet**	**Main outcomes**
*Vitis vinifera* (Grape)	Men and women (20–60 years old) obese (58)	Grape powder (4,600 mg/day) for 9 weeks	LDL-c ↓ IL-1β ↑ IL-6 ↑
*Vaccinium macrocarpon* (Cranberry)	Men and women (30–70 years old) obese (59)	Cranberry extract beverage (450 mL/day) for 8 weeks	Glucose regulation ↑ HDL-c ↑ Serum insulin ↓ CVD ↓ Inflammation ↓
*Mangifera indica* (Mango)	Women (25–45 years old) obese	Peel powder of mango (1 g/2 × day) for 12 weeks	LDL-c ↓ Triglyceride ↓ HDL-c ↑
*Olea europaea* (Olive)	Women (27 years old) obese (60)	EVOO (25 mL/day) for 9 weeks	HDL-c ↑ BW ↓ Blood pressure ↓ Inflammation ↓ Oxidative stress ↓ Dyslipidemia ↓
*Citrullus lanatus* (Watermelon)	Men and women (18–55 years old) obese (61)	Watermelon fruit (2 cups = 152 g/day) for 4 weeks	BW ↓ Blood pressure ↓ CVD ↓ Blood lipid profile ↑ Antioxidant status ↑
*Ilex paraguariensis* (Yerba mate)	Men and women (35–60 years old) obese (62)	Yerba mate tea (500 mL/2 × day) for 4 weeks	Serum level ↑ HDL-c ↑ Atherosclerotic diseases protection ↑
*Lippia citriodora* and *Hibiscus sabdarifa*	Women (36–69 years old) obese (63)	Combination polyphenol extract (500 mg/day) for 8 weeks	BW ↑ Fat metabolism ↑ Adiposity ↑
*Citrus sinensis* (Orange)	Women (29–43 years old) obese (64)	Orange juice (250 mL/ × day) for 12 weeks	Total cholesterol ↑ LDL-c ↑ Inflammation ↓
*Fragaria ananassa* (Strawberry)	Men and women (20–50 years old) obese (65)	Strawberry powder (2 servings = 160 g/day) for 7 weeks	CVD ↑ Stroke ↑ Diabetes ↑
*Cinnamomum verum* (Cinnamon)	Men and women (40–50 years old) obese (66)	Cinnamon extract (250 mg/2 × day) for 12 weeks	Diabetes ↑ CVD ↑ Free radical ↑
*Helianthus annuus* (Sunflower)	Men and women (18–65 years old) obese (67)	Sunflower seed extract (500 mg/day) for 12 weeks	BW ↑ BMI ↑ Cholesterol ↑ Lipid metabolism ↑

*↑, significant increase; ↔, unchanged; ↓, significant decrease; LDL-c, low-density lipoprotein cholesterol; HDL-c, high-density lipoprotein cholesterol; CVD, cardiovascular disease; WC, waist-circumference; BMI, body mass index; TG, triglyceride; BW, body weight; BG, blood glucose; DM, diabetes mellitus; EVOO, extra virgin olive oil; IL-6, interleukin-6; IL-1β, interleukin-1β*.

## The Main ω-3 PUFAs Sources

The main sources of ω-3 PUFAs, including ALA, EPA and DHA are green leafy vegetables, seaweed, seeds, nuts, vegetable oils, fish and fish oils (68–77). The vegetable and fish origin ω-3 PUFAs are summarized in Table 3.

**Table 3:** Contents of n-3 PUFAs and their vegetable and fish sources used in human food.

**Source**	**Food**	**ω-3 PUFAs (%)**			**References**
		**ALA**	**EPA**	**DHA**	
Vegetable	*Moringa oleifera* (flower, pod, leaf)	18.8–54.3	0	0	(68)
	*Brassica* spp.	7.0–20.0	0	0	(69)
	*Lactuca sativa* (baby-leaf)	44.0–55.0	0	0	(77)
	*Solanum* spp. (leaf)	50.0–54.0	0	0	(70)
	Flax and chia seed	22.8	0	0	(69)
Vegetable oil	*Linum usitatissimum* (seed)	53.0–58.3	0	0	(69)
	*Brassica* spp. (seed)	6.8–20.2	0	0	(69, 75)
	*Glycine max* (seed)	6.0–15.9	0	0	(69)
Macroalgae	*Phaeophyta* spp.	0	6.6–14.4	0.8–1.5	(71)
	*Rhodophyta* spp.	0	2.9–27.3	4.9	(71)
Microalgae	*Chroomonas mesostigmatica*	60.3	30.5	1.7	(72)
	*Guillardia theta*	56.7	14.9	3.0	(72)
	*Hemiselmis* sp.	53.2	21.2	5.1	(72)
	*Proteomonas sulcata*	58.5	12.7	12.6	(72)
	*Rhodomonas salina*	48.8	17.2	11.2	(72)
	*Storeatula major*	41.9	16.0	10.0	(72)
	*Teleaulax* spp.	43.3–46.2	23.6–26.0	12.7–14.3	(72)
Fish of freshwater	*Pimelodus* spp.	1.3–3.9	0.4–1.3	1.9–8.2	(73)
	*Ageneiosus brevifilis* (Palmito)	0.9	0.7	8.7	(73)
	*Aspius aspius* (Asp)	2.2	2.6	5.2	(74)
	*Barbus barbus* (Common brarbel)	3.4	2.9	5.6	(74)
	*Acipenser ruthenus* (Sterlet)	4.3	2.9	3.8	(74)
	*Esox lucius* (Northern pike)	2.6	1.6	7.6	(74)
Fish of marine water	*Caranx hippos* (Crevalle jack)	0	3.1	17.6	(74)
	*Thunnus thynnus* (AB tuna)	0	4.8	32.5	(76)
	*Scomberomorus maculatus* (AS mackerel)	0	5.6	12.6	(76)
Fish oil	*Sardine pilchardus* (sardine)	0	10.1	10.7	(69)
	*Brevoortia tyrannus* (menhaden)	0	13.2	8.6	(69)
	*Salmon* spp. (salmon)	0	13.0	18.2	(69)
	*Gadus morhua* (cod liver)	0	6.9	11.0	(69)

*PUFAs, Polyunsaturated fatty acids; ALA, α-linolenic acid; EPA, eicosapentaenoic acid; DHA, docosahexaenoic acid*.

ALA is abundantly obtained in vegetable foodstuff and microalgae (7–94%) followed by vegetable oils (6–58%) and freshwater fish (1–4%) (69, 70, 73–77). While EPA and DHA are the majority in fish oil (7–13% and 9–18%), marine fish (3–6% and 13–33%), microalgae (13–31% and 2–14%), macroalgae (3–27% and 1–5%), and fish of freshwater (0.4–3% and 2–9%) (69, 71–74, 76).

**Figure 2:**
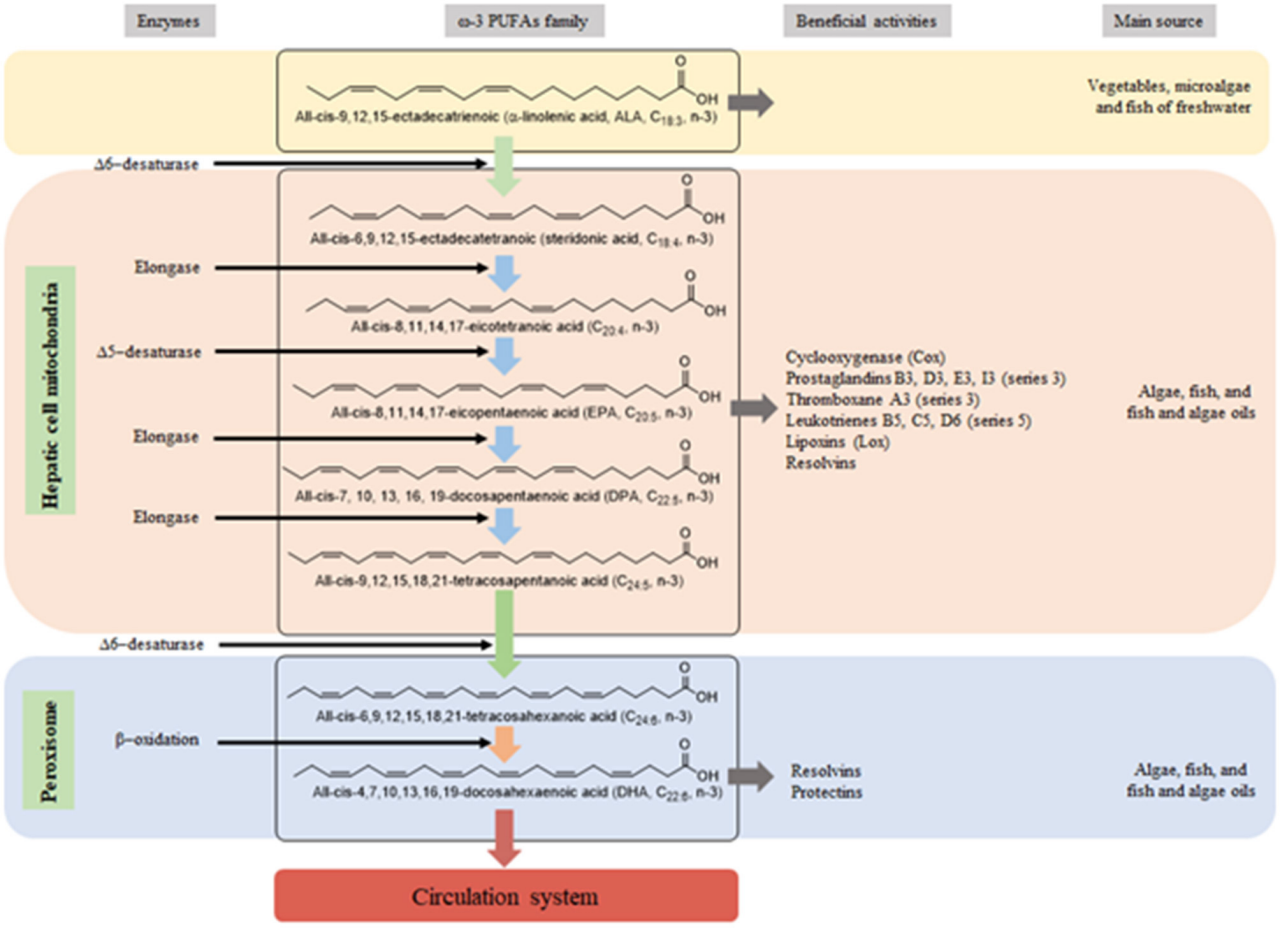
Biosynthesis pathway of very-long-chain polyunsaturated fatty acids (LC-PUFAs) and very long-chain fatty acids (VLCFAs) in the human body starting by the α-linolenic acid (ALA) obtained from the diet. The LC-PUFAs and VLCFAs biosynthesis process occurs in hepatic cell mitochondria and peroxisome. These acids reach the bloodstream, which are conducted to different body parts for health benefits.

The ω-3 PUFAs and ω-6 PUFAs are essential fatty acids (cannot be biosynthesized by the mammalian body, including humans) are required from the diet (78, 79). In the human body, through to physiology mechanism reactions, which ALA is converted to long chain PUFAs (LC-PUFAs, fatty acids ≤ C20) and very-long-chain fatty acids (VLCFAs, fatty acids ≥ C22) (78, 79), which the ALA converted rate to EPA and DHA is 5–8% (80). The biosynthetic process of VLCFAs production, starting by ALA from the diet to the bloodstream is illustrated in Figure 2.

When consumed and going through several physiological reactions in the body, EPA and DHA present positive effects such as anti-inflammation, vasodilation, bronchodilation and antiplatelet aggregation (78). Beyond, both acids are correlated with cyclooxygenase, prostacyclin, thromboxane, leukotrienes, lipoxins, and resolvins, which play a crucial role in several beneficial physiologic actions (78, 79, 81). The consumption of an ω-3 PUFAs-rich balanced diet, including ALA, EPA, and DHA is correlated with health-improving and decreasing and or preventing obesity and its RMDs, such as adipose tissue fat accumulation, insulin resistance, inflammation, hypertension, atherosclerosis, CVD, CHD, and DM (4, 78, 79).

However, due to the presence of double bond in carbon-3 of methyl end (ω-3), including ALA, EPA, and DHA, ω-3 PUFAs family is susceptible to oxidation by light, temperature, metal ions and microorganism degradation during oil extraction and storage by autoxidation reactions (photochemical and photosensitized oxidation) with 4-Hydroxy-2-hexenal production (82, 83). These reactions result in enzymatic oxidation with increase the production of E-series resolvins from EPA, and D-series Resolvins (DHA), prostaglandins, thromboxanes, leukotrienes, epoxy products (84, 85). Besides, the ω-3 PUFAs decrease in amount during food confections by thermal processing, while in inversely proportion occurs the increasing of degradation and hazard oxidized substances that damage cell membranes (86, 87). The oxidation products are higher in fried, followed by roasted, and boiled foods, which present the same proportion of oxidative products when compared to raw food (88, 89). The frying and roasting food confections release the most oxidative products (4(RS)-4-F4t-NeuroP, 4-Hydroxy-2-hexenal production, and others), which are correlated with obesity, CVD, inflammation, hypertension, and others diseases (82, 83).

Therefore, the application of natural antioxidant compounds such as carotenoids, tocopherols, tocotrienols, phytostanols, phytosterols, and ascorbic acid are recommended due to their symbiotic and synergistic interactions decrease oxidation and thermal degradation, prolonging the shelf life of ω-3 PUFAs during the period of storage (90–92).

## Dietary EPA and DHA Diets Benefits on Obesity and its RMDs

Diets consumption rich in vegetables and fish and their by-products are correlated with reducing obesity and its RDMs effects for presenting ALA, EPA, and DHA in their composition (4, 78, 79), and for animal models are summarizing in Table 4.

**Table 4:** Effects of EPA and DHA intake on obesity and related metabolic diseases outcomes in animal models.

**Host**	**Diet**	**Main outcome**
Rats Wistar (6 weeks old) overweight male (93)	EPA ethyl ester of cafeteria diets (1,000 mg/kg/day) for 5 weeks	Body weight ↓ Adipose tissue ↓ Inflammation ↓ Insulin resistance ↓
Rats JCR:LA-cp (3 weeks old) obese male (94)	EPA (5,300 mg) + DHA (9,400 mg/kg/day) for 3 weeks;	Body weight ↓ TG ↓ LDL-c ↓ HDL-c ↑
Rats Wistar (8 weeks old) liver triacylglycerol and insulin resistance male (95)	Fish oil: EPA (328 mg) + DHA (440 mg)/kg/day) for 4 weeks	Hepatic β-oxidation ↑ Hepatic lipogenesis ↓
Mice C57BL/6J (5 weeks old) metabolic syndrome male (96)	Fish and algal oils EPA + DHA oral administrated for 11 weeks 1. EPA (0.03 mg) + DHA (0.06 mg)/kg/day 2. EPA (0.05 mg) + DHA (0.05 mg)/kg/day 3. EPA (0.06 mg) + DHA (0.03 mg)/kg/day	Body weight ↓ LDL-c ↓ Steatosis ↓ Inflammation ↓ TG ↓ TC ↓
Mice C57BL/KsJ-lepr^db^/lepr^db^ (7 weeks old) obese and DM male (97)	EPA (15 mg) + DHA (8 mg)/g/day) for 6 weeks	Adipose tissue ↓
Mice Elovl2 -/- weight gain (98)	Low sucrose + DHA (10,000 mg/kg/day) for 4 weeks	BW ↓
Mice Elovl2 -/- or Wilde-type weight gain (98)	High sucrose + DHA (10,000 mg/kg/day) for 4 weeks	BW ↑
Mice C57BL/6J (6 weeks old) obese male (99)	HFD-EPA (2 mg) + DHA (5 mg)/g/day for 8 weeks	Adipose tissue ↓ Inflammation ↓
Rats Sprague-Dawley (3 weeks old) obese and insulin resistance male (100)	ω-3 + ω-6 PUFAs (83,000 +83,000 mg/kg/day) for 16 weeks	Blood lipid ↓ Body and visceral fat ↓ Glucose tolerance and insulin sensitivity ↑ Pro-inflammatory cytokines ↓
Mice C57BL/6J (3 weeks old) metabolic syndrome male (101)	ALA (92 mg/kg/day) for 10 weeks	Positive hepatic expression ↑ Metabolic parameters ↑ Glycemic parameters ↑
Rats Sprague-Dawley (3 weeks old) inflammation bowel male (102)	LA + ALA (2 g + 1 g/100 g/day) for 12 weeks	Colonic inflammation ↓ Colon length ↑ Pro-inflammatory cytokines ↓ Colon ω-3 PUFAs ↑
Rats Wistar (3 weeks old) metabolic syndrome male (103)	Supplement marine algae *Phaeodactylum tricornutum* (EPA =33 mg/g/day) for 8 weeks	BW ↓ Fat mass ↓ Inflammation ↓ Insulin resistance ↓ TC ↓ Triacylglycerol ↓ Leptin ↓
Mice C57BL/6J (6 weeks old) hepatic steatosis and metabolic syndrome male (104)	Fruits and vegetable powder mixed (EPA = 340 mg/g) for 20 weeks	Weight body ↓ Hepatic steatosis ↓ Inflammation ↓ Blood and liver ceramides ↓

*↑, significant increase; ↓, significant decrease; LA, linoleic acid; ALA, linolenic acid; EPA, eicosapentaenoic acid; DHA, docosahexaenoic acid; ω-3 PUFAs, omega-3 polyunsaturated fatty acids; HFD, high-fat diet; LDL-c, low-density lipoprotein cholesterol; HDL-c, high-density lipoprotein cholesterol; TC, total cholesterol; TG, triglyceride; DM, diabetes mellitus; BW, body weight*.

Obesity and its reduction in RMDs have been reported in animal studies that consumed for 3–20 weeks EPA from vegetable/fruit and cafeteria diets (33 mg/g/day to 1,000 mg/kg/day), EPA mixed with DHA (2–5,300 mg/g and 3–9,400 mg/g/day), ALA (92 g/kg/day), ω-3/ω-6 (1:1), and linoleic acid (LA) plus ALA (2:1) (93, 94, 99, 100, 102–104). These positive effects observed are correlated with ω-3 PUFAs that improve and repair several organs for normal function linked to hepatic organ for better lipogenesis, insulin resistance, lipid homeostasis, adipocytes function, β-oxidation, and increasing leptin and adiponectin production, pro-inflammatory mediators reducing from LA and arachidonic (AA) acids (78, 79, 81). However, some studies reported a discrepancy effect of ω-3 PUFAs to diabetes, cholesterol, plasma glucose (105), overweight and obesity (106), inflammatory cytokines (107), cardiovascular diseases, and others (108). These ω-3 PUFAs fail results can be associated with its preparation, doses quantity, administration duration period, subject target, statistics, and other factors (109, 110). Therefore, ω-3 PUFAs regular consumption is recommended due to numerous studies that demonstrated strong positive effects against several metabolic diseases in animal models and human subjects, as summarized in Table 5.

**Table 5:** Effects of EPA and DHA intake on obesity and its related metabolic diseases outcomes in human subjects.

**Host**	**Diet**	**Main outcome**
Men and women DM (57–68 years old) (111)	Flaxseed powder ω-3 PUFAs–ALA-rich (5 g/2 × day) for 4 weeks	HDL-c ↑ LDL-c ↓ TC ↓ Triglycerides ↓
Men and women hypercholesterolemic (36–65 years old) (112)	LA (20 or 40 g) + ALA (10 g)/day for 1 week	TC ↓ LDL-c ↓ Triglycerides ↓ CVD risk ↓ Inflammation ↓
Men and women CVD (≥ 30 years old) (113)	EPA (600 g) + DHA (1,500 mg)/day from microalgae *Schizochytrium* sp. oil for 4 weeks	LDL-c ↑ HDL-c ↑ LDL/HDL ↔ CVD ↔
Men and women obese and DM (≥ 85 years old) (114)	EPA (1,800 mg/day) in capsule for 12 weeks	BMI ↓ Insulin ↓ LDL-c ↓ HDL-c ↓ TC ↓ TG ↓
Men and women major coronary artery disease (mean 62 years old) (115)	EPA (600 mg/3 × day) for 5 years	DM ↓ Hypertension ↓ LDL-c ↓ HDL-c ↓ TG ↓
Men and women hypercholesterolemic ≥ 6.5 mmol/L (≥ 40 years old) (116)	EPA (300 mg/3 × day) capsuled for 5 years	Stroke ↓ LDL-c ↑ HDL-c ↓ TG ↓
Women (8–20 weeks gestation) obese (≥ 27 years old) (117)	EPA (800 mg) + DHA (1,200 mg)/day for 25 weeks	Inflammation ↓
Men and women (28–60 years old) hypertensive and/or diabetic (118)	EPA (300 mg) + DHA (200 mg)/day capsuled for 8 weeks	Inflammation ↔ TC ↔ TG ↓ BG ↓
Women pre-menopausal elevated triglyceride (<18 or > 40 years old). (119)	Tuna oil DHA (135 mg) + EPA (35 mg)/day for 8 weeks	TG ↓ Blood pressure ↓ HDL-DHA ↑ LDL-DHA ↓ VLDL-TG ↓

*↑, significant increase; ↓, significant decrease; ↔, unchanged; BMI, body mass index; BG, blood glucose; EPA, eicosapentaenoic acid; DHA, docosahexaenoic acid; LDL-c, low-density lipoprotein cholesterol; HDL-c, high-density lipoprotein cholesterol; VLDL, very low- density lipoprotein; TC, total cholesterol; TG, triglyceride; DM, diabetes mellitus; CVD, cardiovascular diseases; ALA, α-linolenic acid; LA, linolenic acid; ω-3 PUFAs, omega-3 polyunsaturated fatty acids*.

Furthermore, lowering obesity and its RMDs were observed for human subjects daily administered 2 ×2 g of flaxseed powder, as well as in proportion of 4:1 and 2:1 of LA and ALA for 1 and 2 weeks (111, 112), EPA daily dosed 3 ×300 mg or 3 ×600 mg, dose of 1,800 mg during 12 weeks and 5 years (114–116), and doses of EPA and DHA during 8–25 weeks in proportions of 1:1.5, 1.5:1, and 1:4 (117–119).

## Polyphenols and ω-3 PUFAs Mechanisms on Obesity and its RMDs

**Figure 3:**
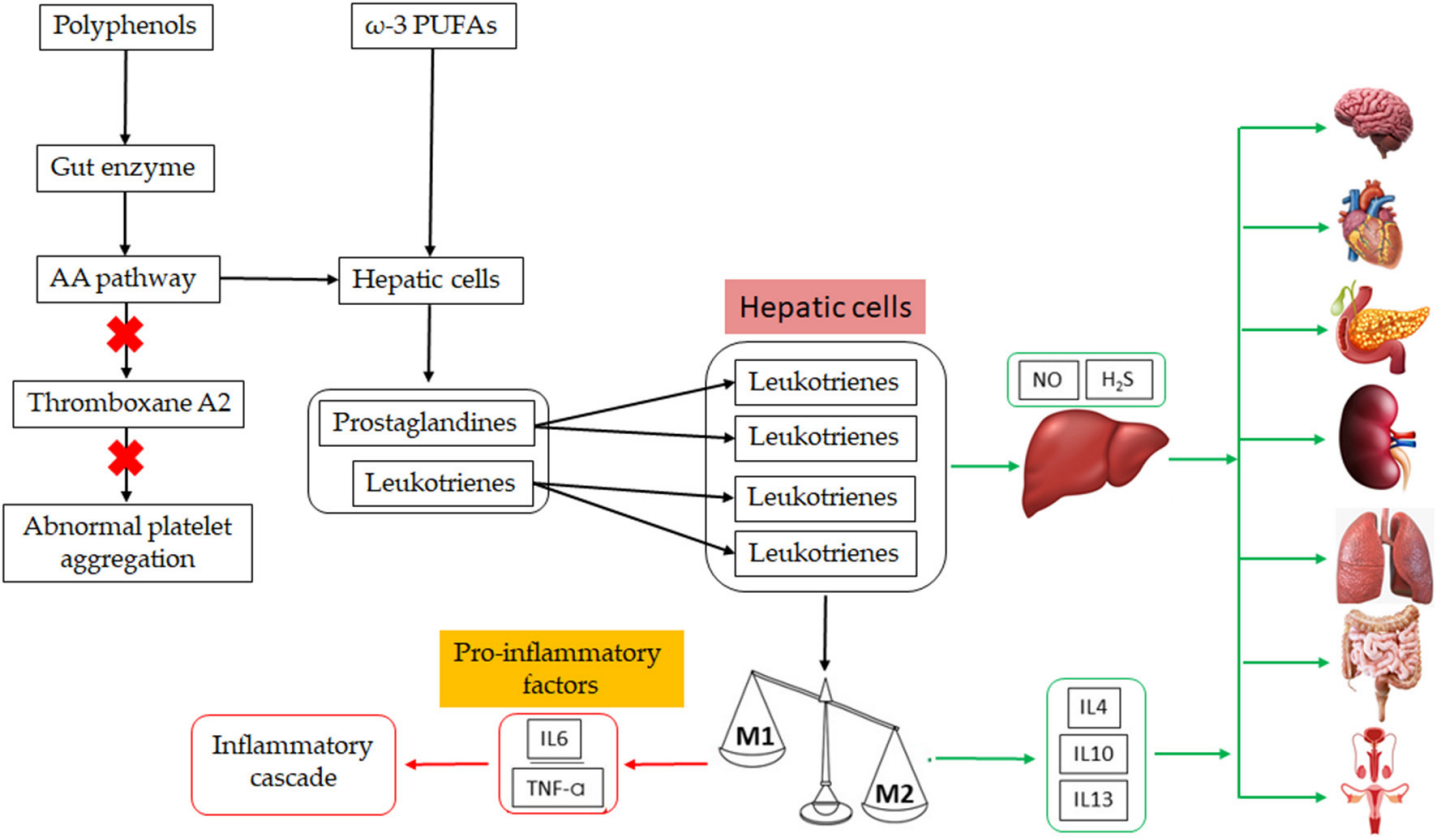
Mechanism involved in an inflammatory condition and its resolution using ω-3 PUFAs and polyphenols dietary. The action of products from PUFAs metabolization (hepatic biosynthesis or tissue under inflammation), lipoxins, resolvins, protectins, and maresins on macrophage profile change and the endothelial cells. As a result, there are anti-inflammatory interleukins, nitric oxide (NO) and hydrogen sulfite (H_2_S) being produced, which will provide the resolution and tissue regeneration. Products from polyphenols metabolization are also connected with this anti-inflammatory pathway to several organs in the body. M1, type 1 macrophages; M2, type 2 macrophages; IL, interleukin; TNF-α, tumor necrosis factor-alpha. Green lines mean resolution of the inflammatory process and red lines mean the uncontrolled inflammatory process leading to an inflammatory cascade.

Increasing of obesity and its RMDs are already observed from childhood to elderly individuals and have become a public health problem in modern society (120, 121). A practical alternative against obesity and its RDMs in humans can be associated with diet-rich in polyphenols and ω-3 PUFAs in composition, including their by-products (112). In the body, polyphenols and ω-3 PUFAs (DHA and EPA) physiologically act protecting and inhibiting cascade inflammatory reaction processes that can evolve into obesity, diabetes, CVD, hypercholesterolemia, and others metabolic diseases (122, 123). Thus, mechanisms that polyphenols and ω-3 PUFAs are involved in the body, which are crucial to prevent several metabolic diseases, which can be used as adjuvant therapy, are summarized in Figure 3.

In the liver, PUFAs are metabolized and converted into prostaglandins (PGE2) and leukotrienes, which reach the inflammation site being converted into lipoxins, resolvins, protectins, and maresins, which will stimulate type 2 macrophages more so than the type 1 kind, leading to the production of anti- inflammatory interleukins (124, 125). Likewise, polyphenols are absorbed in the intestine after being hydrolyzed by intestine enzymes and the host's microbiota (126). Then, the resulting molecules can interact with free radicals and inhibit enzymes involved in the AA pathway, modulating the inflammatory response and blocking the AA pathway (14). Besides that, endothelial cells are also being stimulated by both products from polyphenols and ω-3 PUFAs metabolization to produce NO and H_2_S in the first case, which will aid the resolution of the inflammatory situation and the tissue regeneration, or trigger signaling cascades by interacting with cell membrane receptors such as vascular endothelial growth factor (VEGF) or blocking p-AKT, NF-κB, and MMP-9 activities (122, 127).

The mechanisms involved in balancing the inflammatory process are the change of the phospholipid fatty acid composition of the cell membrane, inhibition of the NF-κβ activation, thus reducing the expression of pro-inflammatory genes and production of resolving mediators by macrophages (122).

## Consumer Behavior Changes on Obesity and its RMDs

Choosing daily healthy food type intake is the chief component and managed by humans to improve their own and all family healthy lifestyle (128). Among the several factors of healthy lifestyle or prevalence of obesity and its RMDs can be associated with regular or irregularly and healthy or unhealthy daily food consumed in each meal (22, 129). In addition, it may also be associated with the lower purchase price of unhealthy foods on the market compared with healthy ones, whose edible parts (leaves, peel, flesh, seeds, and others) are wasted in homes, restaurants and other food enterprises due to their lack of nutritional knowledge (130, 131). In addition, also it is known that refined sugar is often always added to edible vegetables, fruits, natural juices and other by-products and other beverages, which can be associated with obesity, overweight, CVD, and other metabolic diseases prevalence (132).

**Figure 4:**
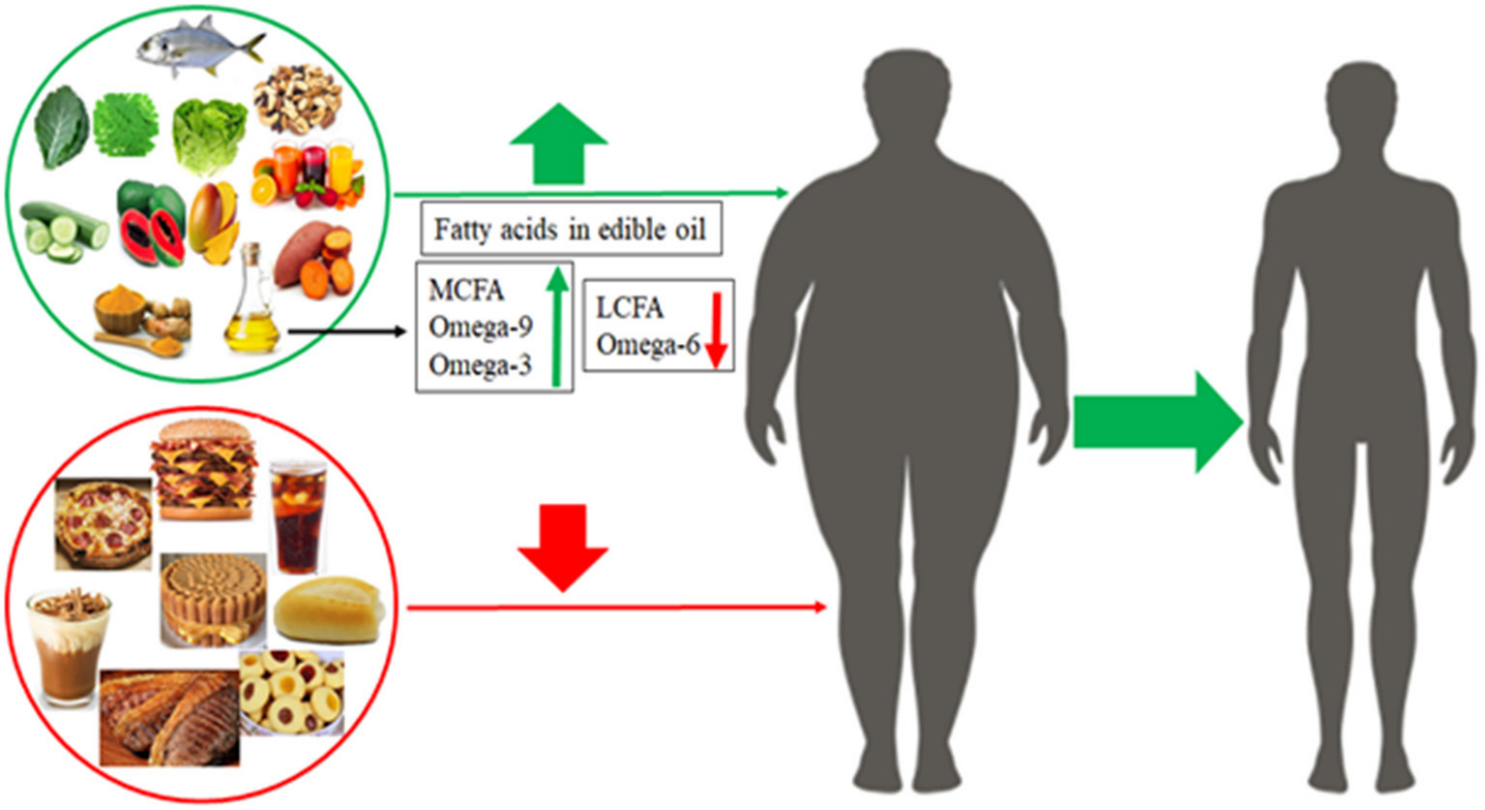
Healthy food (green line) intake reduce obesity to normal conditions, while unhealthy food (red line) conduces to obesity and its related metabolic diseases.

Thereby, Figure 4 summarizes food types that improve healthy life (green line), which oil rich in ω-3 PUFAs, oleic acid and short-chain fatty acid are widely recommended (4). Daily at least 400 mg of natural and/or native fruits and vegetables (133), while weekly 3 ×150 g of fish are recommended (134). Furthermore, fruits, vegetables and fishes are natural sources of macro- and microelements, vitamins, resistant nutrients, free sugars and fibers, which play a crucial role in microbiota balance, satiety, gut health and act as antioxidants in the body, improving and/or impeding obesity and others prevalent metabolic diseases (135–137).

Paradoxically, nowadays, meals rich in vegetables and fruits are associated with poor and traditional peoples, while meat and sweetened ones are associated with rich and modern life (24, 138, 139). The consumption of foods marked by the red line (Figure 4) must be reduced, because they are sweetened and fatted, including long-chain saturated fatty acids (mainly myristic and palmitic acids), ω-6 PUFAs and industrialized trans-fatty acids present high amounts of calories in their composition, which are primarily associated with obesity and its prevalent RDMs (134).

Hence, for human behavior changes, joint activities between Universities, Research Centers, Health Ministries, and others will be legally necessary constitution of Departments that could be responsible by outline joint projects and approaches for health promotion through seminars, and lectures to implement in schools (Primary and Secondary), enterprises and families to promote healthy food cooking, sale, and intake to pave the way to reduce obesity and its RMDs prevalence (140–144).

## Conclusion

The consumption of vegetables, fruits, seed and fish and/or supplements rich in polyphenols and ω-3 PUFAs is widely correlated with reducing of obesity and its related metabolic diseases prevalence. Thus, for behavior change, it is necessary to draw out a joint projects of research institutions and the Health Ministries to schools, enterprises and families to promote healthy food intake to reduce obesity and its related metabolic diseases.

## Author Contributions

TS, DM, VZ-P, DB, AP, and RG conceptualized the topic, researched and analyzed the literature, wrote the manuscript, and including interpretation. PF, GM, PH, MV, RF, EC, and VN contributed with draft and interpretation and revised the manuscript critically for intellectual content. All authors have read and approved the final version of the manuscript, ensure the accuracy and integrity of the work, and agree to be accountable for all appearance.

## Funding

This research was funded by Federal University of Mato Grosso do Sul (UFMS) and Coordination of Higher Education Personnel Improvement (CAPES)-Portaria 2016/2018. This study was financed in part by the CAPES-finance code 001. The study was also supported by research grants from the National Council for Scientific and Technological Development (Conselho Nacional de Desenvolvimento Científico e Tecnológico-CNPq).

## Conflict of Interest

The authors declare that the research was conducted in the absence of any commercial or financial relationships that could be construed as a potential conflict of interest.

## Publisher's Note

All claims expressed in this article are solely those of the authors and do not necessarily represent those of their affiliated organizations, or those of the publisher, the editors and the reviewers. Any product that may be evaluated in this article, or claim that may be made by its manufacturer, is not guaranteed or endorsed by the publisher.
